# Hunting for microsatellite instability in long-read data with Owl

**DOI:** 10.1371/journal.pcbi.1014423

**Published:** 2026-09-02

**Authors:** Zev Kronenberg, Byunggil Yoo, Khi Pin Chua, Mark J. P. Chaisson, Lisa Lansdon, William J. Rowell, Guilherme de Sena Brandine, Jocelyne Bruand, Egor Dolzhenko, Kobe Ikegami, Jay Sarthy, Kie Kyon Huang, Patrick Tan, Shruti Bhise, Everett Fan, Mark Mendoza, Emily O’Donnell, Tomi Pastinen, Elizabeth R. Lawlor, Scott N. Furlan, Midhat S. Farooqi, Michael A. Eberle

**Affiliations:** 1 PacBio, Menlo Park, California, United States of America; 2 Children’s Mercy Hospital, University of Missouri-Kansas City School of Medicine, Kansas City, Missouri, United States of America; 3 University of Southern California, Los Angeles, California, United States of America; 4 Norris Comprehensive Cancer Center, University of Southern California, Los Angeles, California, United States of America; 5 Fred Hutch Cancer Center, Seattle, Washington, United States of America; 6 Duke-NUS Medical School, Singapore, Singapore; 7 Genome Institute of Singapore, Singapore, Singapore; 8 Precision Health Research Singapore, Singapore, Singapore; 9 Ben Towne Center for Childhood Cancer and Blood Disorders Research, Seattle Children’s Research Institute, Seattle, Washington, United States of America; 10 Department of Pediatrics, University of Washington, Seattle, Washington, United States of America; University of Helsinki: Helsingin Yliopisto, FINLAND

## Abstract

Microsatellite instability (MSI) is a key biomarker of mismatch repair deficiency and response to immunotherapy, yet most existing genomic detection methods are optimized for short-read sequencing and rely on a panel of homopolymer markers, limiting the ability to characterize genome-wide and motif-specific patterns of instability. Here we present *Owl*, a bioinformatic tool for quantifying MSI from long-read (PacBio) genomic data. Owl leverages a genome-wide marker set of more than 140,000 microsatellite repeats ranging from 1–6 bp in length to measure MSI across a phased genome. Using a wrap-around alignment algorithm, *Owl* constructs repeat-length distributions at each marker site and flags somatic instability using the coefficient of variation. We applied *Owl* to screen for markers with stable coverage, phasing, and baseline variation across 131 diverse genomes from the Human Pangenome Reference Consortium, where *Owl* scores ranged from 1.4% to 5.4% of markers exceeding the instability threshold. When applied to cancer cell lines and one diffuse astrocytoma tumor-normal pair, *Owl* identified six MSI genomes with 10–27% unstable markers and showed close concordance with an Illumina *DRAGEN* MSI assay for the astrocytoma sample. Motif-level analyses revealed shared enrichment of short homopolymer and dinucleotide (A- and AT-rich) repeats across MSI cancers. *Owl* is implemented in Rust and integrated into the PacBio HiFi Somatic workflow, providing a scalable framework for MSI analysis from long-read sequencing focused on repeat instability specifically in tumor samples.

## Introduction

Deficiencies in DNA repair pathways give rise to somatic mutations ranging from small variants (SNVs and indels) [1] to large-scale structural variants [2] that collectively drive cancer progression. Tracking deficiencies in the DNA repair pathway is typically done indirectly through several genomic metrics. The first is Tumor Mutational Burden (TMB), which measures the accumulation of somatic Single Nucleotide Variant (SNVs) and small insertions and deletions (indels). The second, Homologous Recombination Deficiency (HRD), is a compound metric that tracks the loss of heterozygosity, large-scale state transitions, and telomeric imbalance. Lastly, Microsatellite Instability (MSI) is when somatic insertions and deletions accumulate in simple repeats [3]. Although these measures of genomic instability and scarring do not directly pinpoint the causative DNA repair defect, they remain critical biomarkers for cancer diagnosis and treatment planning [4–6].

MSI cancers typically arise through loss of function mutations in one of several key Mismatch Repair (MMR) genes, including *MLH1*, *MSH2*, *MSH6*, and *PMS2* [7]. Lynch syndrome, for example, is an autosomal dominant hereditary cancer predisposition caused by germline mutations in the MMR genes, commonly *MLH1* and *MSH2* [8]. In contrast, sporadic (non-syndromic) MSI cancers acquire somatic inactivation of these same repair genes in a classical “two-hit” manner. Beyond direct mutations, epigenetic silencing of *MLH1* through promoter hypermethylation is a well-established mechanism of MMR deficiency and a major driver of tumorigenesis in sporadic MSI colorectal and endometrial cancers [9]. MMR driven cancers (including colorectal, endometrial, gastric and ovarian cancers) frequently exhibit high MSI [10]. Although less common, MSI tumors have also been reported in prostate, pancreatic, and brain cancers [11].

Detection methods for microsatellite instability (MSI) from the genome include PCR, Sanger sequencing, and short-read sequencing (SRS) approaches [12]. Genomic assays typically target loci containing simple repeats, such as the BAT-25 and BAT-26 markers [13], which are homopolymer tracts located within introns of *KIT* and *MSH2*. MSI is typically defined by >30% of classical PCR markers loci showing elevated rates of variability [14]. Although efforts to standardize biomarker selection for high-throughput sequencing are increasing [15], existing frameworks such as the Bethesda guidelines [16] and their associated marker panels were developed for PCR-based assays and have not scaled to whole-genome sequencing (WGS). Ideal WGS-compatible markers should be stable across human populations, robust to technical artifacts, easily profiled using whole-genome (WGS) or targeted exome sequencing (ES), and particularly sensitive to replication errors characteristic of mismatch repair (MMR)-deficient cells.

The MSI status of tumors is important to determine the immunotherapies most likely to be effective [10]. For example, frameshift mutations can generate novel peptides, or neoantigens, that are recognized as foreign by the immune system and tumor-infiltrating T cells can detect these neoantigens and mount an immune response. However, this immune response is often suppressed by immune checkpoint pathways such as the PD-1/PD-L1 axis. Checkpoint inhibitor antibodies, including those targeting PD-1, release this inhibition and allow T cells to mount a stronger antitumor response. In patients with MSI tumors, these therapies have been associated with markedly improved clinical outcomes. Sahin and colleagues provide a comprehensive review of MSI-targeted immunotherapy [14].

There have been several bioinformatic tools developed to detect MSI in short-read sequencing (SRS) data, encompassing both genome (GS) and exome (ES) sequencing. Modern SRS-based assays typically classify tumors as MSI when approximately 10–20% of evaluated loci are unstable, with the exact cutoff varying by algorithm and calibration dataset. Commonly used tools include *MSIsensor* [6], *MANTIS* *[*17*]*, *MSIsensor-pro* [18], *mSING* [19], and *DRAGEN*, most of which have been independently benchmarked by Anthony and Seoighe [20], who found that these methods generally perform better on ES data than on GS data. A key challenge in detecting MSI from SRS data is distinguishing somatic mutations from germline heterozygosity, since SRS data are not phased. To address this limitation, some tools compare repeat-length distributions between paired tumor and normal samples. However, matched normal samples are not always available and add to assay cost, and SRS inherently struggles to resolve long or complex repeats due to mapping ambiguities.

Long-read sequencing (LRS), including PacBio HiFi and Oxford Nanopore Technologies, offers several advantages for MSI detection compared to SRS. Long reads span nearly all microsatellite regions, enabling accurate repeat localization through improved mapping, and long-read phasing provides haplotype-level resolution of repeat variation within microsatellites. However, LRS has historically been limited by higher indel error rates and greater cost relative to SRS. As LRS becomes more practical (i.e., higher throughput and lower sequencing cost), it is being adopted for tumor analysis of structural variation, direct profiling of DNA methylation, and full-length transcript sequencing [21–23]. A complete analysis of tumors sequenced with long reads requires software tools developed specifically to work with this data type.

**Fig 1:**
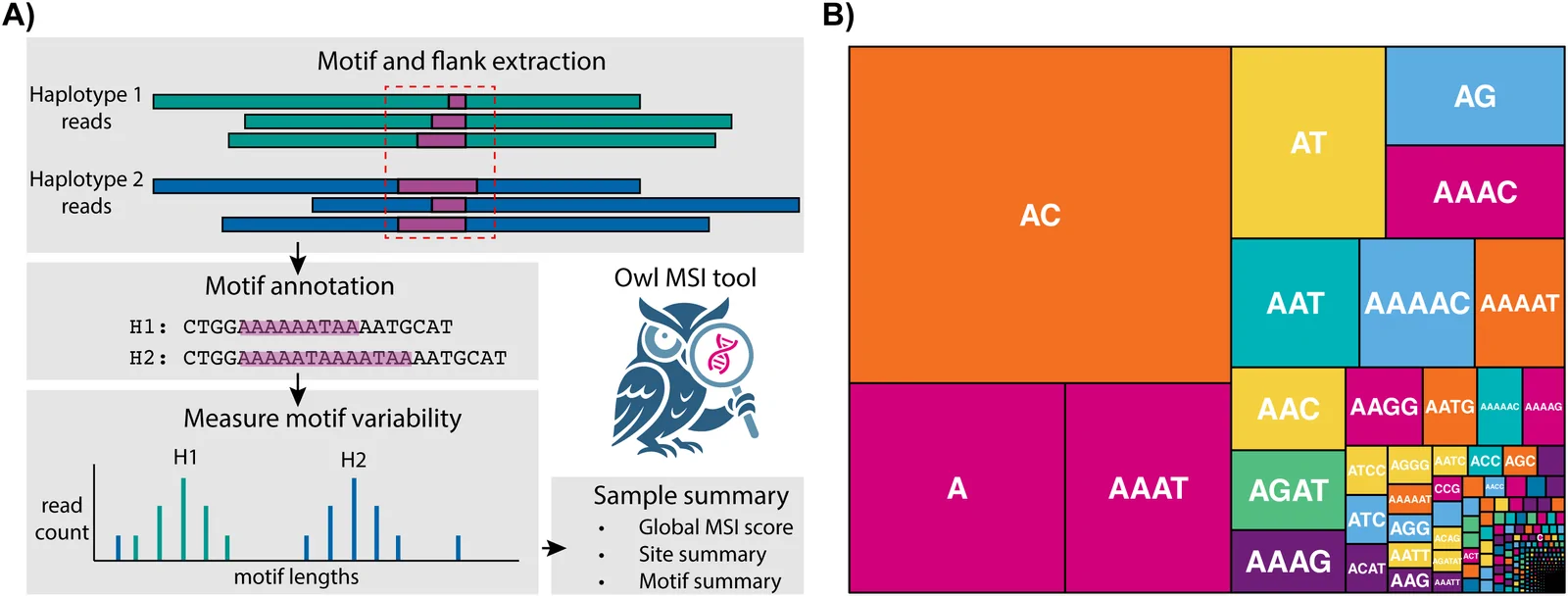
Owl workflow and marker set. **A)** The *Owl* workflow starts with a mapped and phased HiFi genome. In the first stage (profile), reads spanning predefined marker sites are extracted, including flanking bases. Repeats are then annotated in each read using a wrap-around dynamic programming algorithm to determine the matching motif repeat length. These repeat lengths are then grouped by haplotype, and for each marker, the mean, standard deviation, and coefficient of variation are calculated. In the second stage (*score*), *Owl* summarizes MSI across all markers and produces human-readable output files for both profiling and scoring. **B)** Treemap of repeat motif frequencies in the GRCh38 marker set used by *Owl*. Each box represents a unique (lexicographically minimized) repeat motif, with area proportional to its number of genomic sites. The plot shows motif sequences only; repeat lengths are not depicted.

In this study, we present *Owl* (Fig 1A), a bioinformatic tool for determining whether a sample exhibits MSI or microsatellite-stable (MSS) characteristics. *Owl* takes advantage of LR WGS, including precise repeat mapping and read-level phasing, to quantify the fraction of marker sites that exhibit MSI at haplotypic resolution. We examine variation at these marker sites across a diverse panel of human population samples (HPRC), cancer cell lines, and real-world tumor specimens to identify the most reliable indicators of MSI. Our results show that *Owl* provides highly accurate detection of MSI and establishes a robust framework for characterizing genomic instability in cancer.

## Design and implementation

*Owl* consists of two primary modules: profile, which processes read alignments; and score, which calculates and summarizes MSI at the sample level (Fig 1A). The profile module operates on aligned and phased HiFi reads together with a bed file of repeat regions and their corresponding motifs. For each marker, the repeat motif is annotated within reads using wrap-around alignment (see S1 File). Reads are then grouped by haplotype, and for each locus, the coefficient of variation (CV) of repeat length is calculated, serving as the principal per-locus measure of instability used throughout *Owl*. The score module then aggregates the per-locus CV values genome-wide, reporting the percentage of markers that exceed the predefined CV threshold of five, derived from a control genome (see S1 File). The output also includes summary statistics such as the fraction of phased markers, the proportion of loci exhibiting high CV, and a motif-specific breakdown of results.

*Owl* requires a catalog of repeats in the human reference genome GRCh38 for instability analysis. To generate this reference-based catalog, we curated microsatellite annotations from RepeatMasker tracks available through the UCSC Genome Browser [24]. We selected simple 1–6 bp repeats spanning 20–100 bp with sequence identity greater than 97%. A stringent motif-to-reference alignment identity cutoff was required because the *Owl* alignment algorithm was not optimized for divergent or interrupted repeat copies. Markers were restricted to canonical chromosomes (S1 Fig) (excluding chromosome Y). This produced 164,374 repeat sites including 11.6% homopolymers, 45% dinucleotide repeats, and 8.6% trinucleotide repeats. There were 340 distinct motifs after lexicographically minimizing rotations and reverse complements of the original 1,573 motifs (S1 Table). Notably, our identity filtering indirectly excluded most C/G homopolymers and di-nucleotides (48 remaining), which are depleted in the human genome due to its ~ 60% AT bias. Methylation and subsequent deamination of C to T [25] frequently disrupt poly-C tracts, leading to higher divergence among C (15%) and G (9%) homopolymers compared to A (5%) and T (7%).

For a marker/technology combination to best detect MSI, it is important that the repeats used do not show excessive signals of instability in control samples that may be caused by technical artifacts. We profiled the 164,374 *Owl* markers in a set of control PacBio HiFi genomes derived from lymphoblastoid cell lines (S2 Table). The dataset included 131 diverse samples from the Human Pangenome Reference Consortium (HPRC) [26]. From this analysis, we removed 17,790 markers that had a high no-call rate or CV score, in the control HPRC data, leaving us with 146,562 high quality markers (see S1 File, Fig 1B). Across these controls, the *Owl* score, which is the percentage of markers classified as highly variable, ranged from 1.39% to 5.39%, with a mean of 2.18% and a standard deviation of 0.51. Weighted by total genomic counts, background instability varied by motif length, with 7.84% for homopolymers, 1.71% for dinucleotides, and 1.11% for trinucleotides. Although C or G homopolymers and CG dinucleotides showed high apparent instability (61% and 29%, respectively), these motifs are extremely rare in the marker set and together account for only 48 sites. Their low abundance makes the instability percentage highly sensitive to sampling noise. Among the 1,287 motifs with more than 10% high across any control sample, only 21 (1.6%) have more than 100 genomic sites per individual. This shows that motifs with high apparent instability are predominantly low-frequency, and their overall contribution to the MSI score is naturally minimized by their low genomic abundance.

## Results

**Fig 2:**
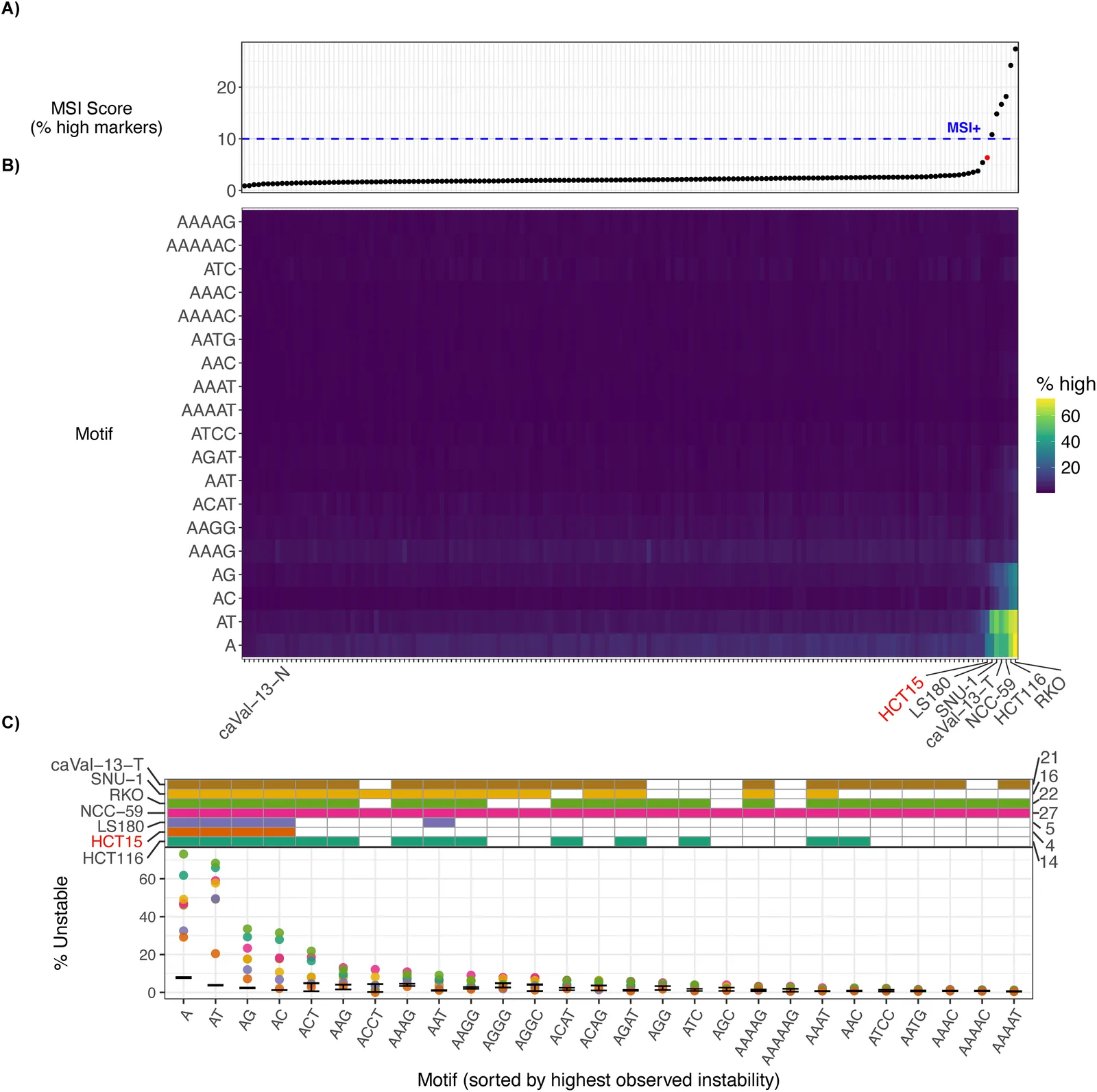
Per-sample MSI scores and motif-specific microsatellite variability. **A)** *Owl* MSI scores for each sample, sorted from low to high. Three colorectal cell lines (RKO, HCT116, LS180), two gastric cancers (SNU-1 and NCC-59), and one diffuse astrocytoma (caVal13) exceed the 10% MSI threshold marked with a blue dashed line. The false negative colorectal cancer cell line (HCT15) is colored red. **B)** Heatmap showing the percentage of high-variability sites per motif, restricted to the most frequent motifs (>500 marker loci). Samples along the x-axis follow the order from the panel above, while motifs along the y-axis are clustered by similarity using hclust. The six MSI samples and the diffuse astrocytoma control (N) are labeled. **C)** Outlier motifs in the six MSI samples, and the one false negative (HCT15 denoted with red text). The top panel marks which sample contains a Bonferroni-corrected p-value (p < 0.05) for a beta-binomial one-sided test. The bottom panel shows, for each motif (lexicographically and rotationally minimized), the percentage of sites with high CV. Each point represents one of the seven samples. The beta-binomial credible intervals (95%) for the HPRC samples are shown as black lines. Only motifs with >100 markers across the genome are shown. The number of motifs reaching significance level, in each sample, is labeled on the right.

Across the full dataset, we analyzed seven Ewing sarcoma cell lines (tumor only), one melanoma cell line with its matched control, four breast cancer cell lines with controls and one without, two lung cancer cell lines with controls and one without, two gastric carcinoma cell lines (tumor only), four colorectal cancer cell lines, and one diffuse astrocytoma with its matched control (S2 Table). Relative to the natural ~1–6% range of instability observed in healthy genomes, six outlier cancer samples showed markedly elevated *Owl* scores ranging from 10-27% (Fig 2A and S2 Table). These MSI samples included three colorectal cancers (LS180, HCT116, and RKO), the diffuse astrocytoma (caVal13-T), and two gastric cancer samples (NCC-59 and SNU-1).

Next, we analyzed baseline motif-specific instability across the 131 HPRC controls (Fig 2B). This analysis was independent of the MSI score and modeled which motifs in the cancer genomes were instability outliers. We modeled the controls using a hierarchical beta-binomial model that pools counts across samples for each motif and generates posterior-predictive p-values to flag motifs whose observed instability exceeds expectation (see S1 File). Across the MSI samples, 33 motifs remained significant after Bonferroni correction (α = 0.05). However, only 27 of these were supported by more than 100 genomic markers. Four motifs (A, AT, AG, AC) were found to be unstable in all six MSI samples (Fig 2B and 2C and S4 Table). The shared motifs suggest that the shorter motifs (1–2 bp) represent the strongest biological signal of MSI. Using a single available tumor-normal pair, caVal-13, a diffuse astrocytoma, we found a negative correlation between motif length and coefficient of variation (CV) scores, with the strongest differences observed in homopolymers and dinucleotide repeats, and only modest changes in trinucleotide motifs (S3 Fig). Among dinucleotides, the AT motif showed the largest average difference between tumor (5.68) and normal (2.65) CV scores. Although homopolymer and dinucleotide repeats often display elevated CVs even in microsatellite-stable samples (Fig 2B), the magnitude of variability was substantially higher in the cancer sample, providing a clear signal differentiation.

To evaluate how MSI calls derived from phased long-read data relate to those from short-read sequencing, we compared instability detected by both technologies. We first examined calls from the *DRAGEN* MSI caller using short-read exome data from the same diffuse astrocytoma sample. *DRAGEN* identified 20% of 12,315 evaluated homopolymer loci as unstable, whereas *Owl* detected 16.67% unstable sites across its long-read marker set (S2 Table). When *DRAGEN* was run in tumor-only mode using a panel of normals as controls, the fraction of unstable loci decreased from 20% to 11%, illustrating how the underlying analysis mode influences MSI estimates in short-read assays. Despite differences in input data and marker sets, both approaches consistently classified the sample as MSI using lower, genome-wide thresholds (approximately 10–20%), reflecting the broader marker coverage relative to legacy PCR-based MSI assays (~30%).

Next, we compared 11 tumor datasets with matched HiFi and Illumina WGS data, including six additional cancer cell lines sequenced for this study (S5 Table). Illumina data were analyzed with *MSIsensor-pro*, and HiFi data were analyzed with *Owl*. For this comparison we used a 10% threshold for both tools. Three samples (LS180, HCT116, RKO) showed elevated MSI scores by both methods (>10%), five samples (TC71, RDES, SKNMC, A673, A549) where both methods agreed on low MSI scores (<10%). The remaining three samples (PDX305, MCF7, HCT15) showed discordant classification between *Owl* and *MSIsensor-pro*. For PDX305 and MCF7, the MSIsensor-pro scores were near the classification threshold, at 10.55 and 11.94**,** respectively. These borderline scores should be interpreted cautiously, particularly because PDX305 is an Ewing sarcoma model, a tumor type not typically associated with MSI-high status, and MCF7 has previously been reported to be MSS [27]. The HCT15 cell line was the only sample with clear evidence that *Owl* classified as a false negative, as is known to be MSI (Vilar et al. 2008). HCT15 had a modest *Owl* score of 6.35, mostly driven by homopolymers (Fig 1A**-**1C). HCT15 has an *MSH6* deficiency which results in a low amount of instability, relative to other MSI samples [28]. Given one false negative in this small sample size we calculate an F1 score for *Owl* of 90.9%.

We assessed the impact of sequencing coverage on phasing and downstream MSI analysis. HiFi data from one MSS sample (NA12878) and one MSI sample (NCC-59) were downsampled to 7.5-20x coverage. For both samples, *Owl* MSI scores decreased with increasing coverage (S4 Fig and S6 Table), consistent with expectations. At lower coverage, only ~40–60% of marker sites were phased (S6 Table), indicating reduced phasing accuracy; resulting haplotype mixing can mimic instability and inflate MSI scores. At moderate coverage, the fraction of phased sites increased to ~91–94%, corresponding to improved phasing accuracy and lower MSI scores. For the MSI NCC-59 sample, the *Owl* score stabilized at ~20x coverage. Together, these results suggest that *Owl* performs optimally at ≥20x coverage.

Using the NCC-59 titration series we evaluated runtime (S7 Table). Runtime increased approximately linearly with sequencing depth, ranging from ~5–9 minutes at 7.5x coverage to ~13–21 minutes at 20x, with low variability across replicates (3–30 seconds). As *Owl* is not multithreaded, runtimes reflect single-core performance and scale predictably with input size.

## Availability

The software is public and distributed under PacBio’s license on github: https://github.com/PacificBiosciences/owl.

## Future directions

We present *Owl*, the first microsatellite instability (MSI) detection tool developed specifically for long-read sequencing data from cancer samples and optimized for PacBio HiFi data. Unlike short-read MSI callers, *Owl* leverages full-length repeat coverage and haplotype phasing to quantify variability in simple sequence repeats across the genome. *Owl* provides a genome-wide instability score that integrates directly into the PacBio HiFi somatic workflow [29]. *Owl* is concordant with *DRAGEN* at the genome-wide level, underscoring the robustness of long-read MSI profiling.

A key advantage of *Owl* is that it can operate using tumor-only samples. Because short-read methods do not phase reads, variation across haplotypes must be analyzed in aggregate, forcing reliance on either a matched normal or a panel of normals to distinguish true somatic microsatellite mutations from pooled haplotypic variation. Likewise, when *Owl* is forced to collapse haplotypes across all markers, it shows substantially inflated repeat-length variation (~28%) compared with the ~ 1% variation observed when phase information is leveraged. However, by preserving haplotype structure, *Owl* can accurately resolve somatic microsatellite changes without matched controls, an advantage that is particularly important in settings where obtaining normal tissue is challenging or not feasible.

*Owl* is also designed to capture motif-specific patterns of microsatellite instability, enabling a more granular view of repeat variation than a single genome-wide score. By comparing each motif’s instability against background rates derived from 131 HPRC control genomes, we found that homopolymer and dinucleotide repeats show the strongest enrichment of instability across all MSI samples, making them the most sensitive reporters of variation in long-read data.

The present *Owl* MSI analyses are limited by the number of long-read cancer genomes currently available, and the comparisons presented here are restricted to a single primary tumor sample. The *Owl* global threshold of 10% was empirically defined using cell line data and may require adjustment in larger cancer cohorts. The per-locus CV score threshold, global MSI threshold, and marker set are all adjustable. With a cohort of primary tumors and orthogonal MSI assay results, a logistic classifier could be trained to provide more refined MSS/MSI classification for HiFi data.

Future development will focus on improved per-locus specificity, expanding support for targeted long-read panels to enable higher-throughput applications, and extending the framework to RNA to explore instability signatures at the transcript level. Layering HiFi methylation data onto this framework may further provide a more integrated view of tumor epigenetic and repeat-instability states. As new methods for somatic phasing become available, these capabilities could also be integrated to better resolve allele-specific instability within tumor genomes.

Together, these results establish *Owl* as a robust framework for MSI detection from long-read data, extending the analytical reach of HiFi sequencing in cancer genomics and providing a foundation for future studies of repeat instability across diverse tumor types.

## Supporting information

S1 Fig Density of marker sites along GRCh38. The density is smoothed in 1Mb windows. The grey density track is for all the marker sites, and red is for marker sites with extreme variability (CV > 20) in one or more analyzed samples. Chromosome Y was excluded from the *Owl* marker set.
(TIF)

S2 Fig Summary of the Gamma distribution fit to NA12878 CV scores. The data were filtered to exclude zero values (no variation), and extremely high values (CV > 20).
(TIF)

S3 Fig A comparison of the Coefficient of Variance (CV) for the CaVal13 paired Tumor/Normal sample. Values with CV scores > 20 were excluded. Each data represents an alleles CV score. The values are binned by the motif repeat unit length. Most of the CV signals differentiating tumor from normal are homopolymers and di-nucleotides.
(TIF)

S4 Fig Effect of sequencing depth on phasing and MSI score. A control genome, NA12878, and MSI gastric cancer cell line NCC-59 were downsampled to 7.5x to 20x coverage, then aligned, variant-called, phased, and tested for MSI. Three replicates were generated at each depth. Points are colored by the percentage of marker sites successfully phased. As sequencing depth increases, a greater fraction of reads are correctly assigned to two haplotypes, and the measured instability decreases, resulting in a lower MSI score. Based on this titration series, 20x coverage is recommended.
(TIF)

S1 Table The composition of the Owl GRCh38 marker set. Each motif has been lexicographically minimized (rotated, reverse complemented, and minima selected). The count is the frequency of the motif.
(XLSX)

S2 Table The Owl summary score for each sample analyzed. For a detailed description of the fields please see documentation (https://github.com/PacificBiosciences/owl#output). The cancer types were manually annotated.
(XLSX)

S3 Table Parametric model fitting for CV thresholding. (XLSX)

S4 Table Top ten outlier motifs for the MSI samples. The columns include the output of the Owl motif summary (https://github.com/PacificBiosciences/owl#output) as well as the confidence intervals from the statistical analysis and the corrected p-value.
(XLSX)

S5 Table MSISensor-pro and Owl comparison. Columns include the source of the data, the MSISensor-pro site counts, %MSI, and %OWL MSI.
(XLSX)

S6 Table Depth titrations. The columns included the output of the owl motif summary (https://github.com/PacificBiosciences/owl#output).
(XLSX)

S7 Table Runtime information, as calculated by Snakemake. (XLSX)

S1 File Supplemental methods [30–38]. Detailed descriptions of the Owl microsatellite instability detection workflow, marker filtering, statistical modeling, comparator analyses, sequencing protocols, and performance benchmarking.
(DOCX)
