# Supplementary material for: Hunting for microsatellite instability in long-read data with Owl
Source: PLoS Comput Biol. 2026 Sep 2;22(9):e1014423. doi: 10.1371/journal.pcbi.1014423 (PMC13585337; doi:10.1371/journal.pcbi.1014423)
Supplement: Supplementary file 1 [file pcbi.1014423.s013.pdf]

Paper Review

Title of the paper: Hunting for microsatellite instability in long-read data with Owl

Manuscript Number: PCOMPBIOL-D-26-00340

Summary of the research

Owl tool is a timely and well-constructed contribution addressing a genuine gap, though validation dataset is limited. Owl is developed to detect microsatellite instability (MSI) from long read sequencing data particularly sequenced with PacBio HiFi. Existing tools are limited to short-read sequencing technology data and rely on small panels of markers; this limits the depth and resolution of MSI analysis.

Owl tool addresses the gap by checking the whole genome for set of over 140,000 MSI locations that are spanning repeats (Motifs) from 1 – 6 base pairs. By differentiating DNA inherited from father and mother it spots s cancer specific mutations avoiding the need of extracting health tissue from the patient. This is very useful when healthy tissue is not available.

Baseline is set using Owl across 131 healthy human genomes and is tested with 19 cancer cell lines and one real brain tumor sample form a patient. Owl identified 5 MSI high cancer samples and showed results closely matched DRAGEN MSI assay. Motif level analysis by Owl reveled a distinct GGAA repeat that is instable among two Ewing sarcoma cell lines. This finding is supported by known biology of EWS FLI1 fusion protein and are not identified by standard MSI assays.

Strengths

1. This tool fills a gap in the long-read sequencing: There is no MSI identification tool for cancer genomics. Although MSIanalyzer is available, it is more specific to 5 loci.
2. Identifying GGAA instability in Ewing sarcoma is novel and can have clinical implications.
3. Baseline is set by using 131 diverse HPRC genomes: - This is significant because it ensures the instability threshold is robust across different human genomes and reduce risk of population biases.
4. Statistical methods like Beta-Binominal model, permutation testing, and Bonferroni used are appropriate.
5. Software is publicly available.

Weaknesses

1. DRAGEN validation is only done on single tumor sample.
2. Validation of Owl tool is not done on primary tumor sample except one clinical sample.

Examples and evidence

Major Issues

1. Owl validation against DRAGEN is only done on single sample, insufficient to establish strong concordance. Including additional paired samples with known MSI that are validated by completely different methods.
2. Data availability from Singapore gastric cancer samples is incomplete.

Minor issues

1. The GGAA finding related to Ewing sarcoma tumor should be validated against few tumor samples to make sure it's not related to a cell culture.
2. CHLA9/CHLA10 relation should be discussed more in the main text rather than limiting to a supplement figure.

Questions for the Authors

1. How does OWL perform at lower sequencing depts? Is there a minimum depth below which performance degrades significantly?
2. What is the computational performance based on the provided data?

Overall recommendation

Accept with Minor to Moderate Revisions.
