# Supplementary material for: Hunting for microsatellite instability in long-read data with Owl
Source: PLoS Comput Biol. 2026 Sep 2;22(9):e1014423. doi: 10.1371/journal.pcbi.1014423 (PMC13585337; doi:10.1371/journal.pcbi.1014423)
Supplement: Supplementary file 2 [file pcbi.1014423.s015.pdf]

Dear Reviewers,

Thank you for your thoughtful review and constructive suggestions. One of the major issues raised was the scarcity of data available for long-read MSI analysis, particularly from primary tumors. This limitation led to some weaknesses in our analysis and in our comparisons of Owl with short-read methods. Although we remain constrained by the number of available samples, especially patient-derived datasets, we made a concerted effort to expand the analysis where possible.

Specifically, we sequenced six additional cancer cell lines that are more appropriate for MSI analysis, including colorectal cancer lines. We then identified matched short-read datasets and used them to compare Owl with short-read MSI methods in a meta-analysis. Ideally, we would have access to additional samples, matched controls, and primary patient data, but we have made the most of the data currently available.

The reviewers' comments and suggestions also encouraged us to examine the GGAA motif instability signal more closely. During this analysis, we discovered that two samples, CHLA10 and TC32, showed conclusive evidence of sample mixing, which led to false MSI scores. We verified this by tracking mitochondrial SNPs that were cross-referenced between the two samples. As a result, we have removed this analysis from the manuscript, which also created space for the additional analyses requested by the reviewers.

We have now added titration analyses, runtime benchmarking, and other requested analyses, which we believe address the reviewers' concerns. Please see the point-by-point response below.

Sincerely,
Zev Kronenberg

Reviewers' comments:

Reviewer's Responses to Questions

Comments to the Authors:
Please note here if the review is uploaded as an attachment.

Reviewer #1:

Examples and evidence

Major Issues

1. Owl validation against DRAGEN is only done on single sample, Insufficient to establish strong concordance. Including additional paired samples with known MSI that are validated by completely different methods.

We agree that concordance studies remain limited, largely due to sample availability and the relatively early stage of long-read cancer method development. Within these constraints, we have made the most comprehensive comparison possible using the samples available to us. Specifically, we extended the comparison between short-read and long-read MSI callers by adding six new HiFi-sequenced samples and including MSIsensor-pro in the comparisons. This expanded analysis provides additional support for the observed concordance while acknowledging that broader benchmarking will require larger cohorts and further maturation of long-read cancer analysis methods.

Please reference the new results paragraph comparing Owl to MSIsensor-pro.

2. Data availability from Singapore gastric cancer samples is incomplete.

Thank you for catching the oversight. The following text has been added:

> ***The two gastric cancer cell line samples:***
>
> *https://ega-archive.org/datasets/EGAD50000002300*

Minor issues

1. The GGAA finding related to Ewing sarcoma tumor should be validated

against few tumor samples to make sure it’s not related to a cell culture.

The GGAA finding was caused by sample contamination (CHLA10, TC32). It has been removed from the manuscript.

2. CHLA9/CHLA10 relation should be discussed more in the main text rather than limiting to a supplement figure.

The GGAA finding was caused by sample contamination (TC32, CHLA10). It has been removed from the manuscript.

Questions for the Authors

1. How does OWL perform at lower sequencing depts? Is there a minimum depth below which performance degrades significantly?

Thank you for this insightful question. We performed a titration experiment using the MSI gastric cancer cell line NCC-59 and the MSS sample NA12878 cell line, with coverage ranging from 7-20x. The results, shown in Figure S6, demonstrate the impact of sequencing depth on both the MSI score and the fraction of phased sites.

Based on these data, we find that ~20x coverage is required to achieve stable phasing, which is necessary for accurate Owl analysis. In practice, most cancer sequencing studies use substantially higher coverage to support robust somatic variant detection, making performance at lower depths less critical.

> *Added text to the results section:*
>
> *For our final experiment, we assessed the impact of sequencing coverage on phasing and downstream MSI analysis. HiFi data from one MSS sample (NA12878) and one MSI sample (NCC-59) were down sampled to 7.5-20x coverage. For both samples, Owl MSI scores decreased with increasing coverage (**Figure S4, Table S6**), consistent with expectations. At lower coverage, only ~40-60% of marker sites were phased (Table S6), indicating reduced phasing accuracy; resulting haplotype mixing can mimic instability and inflate MSI scores. At moderate coverage, the fraction of phased sites increased to ~91-94%, corresponding to improved phasing accuracy and lower MSI scores. For the MSI sample, the Owl score stabilized at ~20x coverage. Together, these results suggest that Owl performs optimally at ≥20x coverage.*

2. What is the computational performance based on the provided data?

We have added that information at the end of the results section and Table S7.

> *Added text to the results section:*
>
> *We additionally evaluated runtime using this titration series (**Table S7**). Runtime increased approximately linearly with sequencing depth, ranging from ~5-9 minutes at 7.5x coverage to ~13-21 minutes at 20x, with low variability across replicates (3-30 seconds). As Owl is not multithreaded, runtimes reflect single-core performance and scale predictably with input size.*

Reviewer #2:

The manuscript introduces Owl, a computational method for identifying microsatellite instability (MSI) with long read sequencing (PacBio) data. The proposed method is well motivated, and the use of haplotype resolved reads provides advantage to traditional short read methods. In addition, the motif-level and regulatory insights enabled by this framework highlight the added value of long-read sequencing beyond conventional MSI classification. Overall, this method is a valuable contribution to current bioinformatics tools for long-read data.

A few minor revisions would strengthen the validation of the tool:
1. The benchmarking is limited to small set of samples against few tools. Although it is discussed that conventional MSI assays on homopolymers might misclassify sarcoma samples with elevated GGAA repeat instability, it would be great to include a figure panel to illustrate cases where Owl outperforms established tools (if short read sequencing data is available on the same samples) and methods (for example, using canonical marker panels only).

We agree, the comparisons against other short-read methods were limited. This was mostly caused by the fact that long-read cancer analyses are relatively new and there are limited public samples. We've sequenced six additional cell lines that are more appropriate for MSI studies (colorectal cancers).

Please reference the new results paragraph comparing Owl to MSIsensor-pro.

2. Since the method relies on phasing, a brief discussion of how lower sequencing depth and reduced phasing quality affect results would increase the clarity. A comment on applicability to other long-read platforms would be helpful.

Thank you for this insightful question. We have now performed a titration experiment using the MSI gastric cancer cell line NCC-59 across coverage levels ranging from 7x to 20x. The results are presented in the new Figure S6, which illustrates the impact of sequencing depth on both the MSI score and the fraction of phased sites.

Based on these data, we recommend approximately 20× coverage to achieve stable phasing, which is required for Owl. In practice, most cancer sequencing applications will use substantially higher coverage to enable reliable detection of somatic variants, making performance at lower depths less critical.

3. In line 405-413, parameters (alpha_m, beta_m, k_m and n_m) should be reformatted as subscripts for consistency.

Done

Reviewer #3:

Kronenberg et al created a tool called Owl for detecting microsatellite instabilities using long read sequencing data. While Owl was used over a diverse panel of cell lines and reported some useful results, there are several limitations and unclear design choices. There also appear to be some inconsistencies between Fig 2B and 2C.

Major points:
The set of marker coordinates was derived from RepeatMasker which I think must have been obtained from short read sequencing (SRS). It appears that Owl limits its analysis to those regions obtained by SRS, while in theory it might use the full power of long read sequencing (LRS). It would be interesting if the authors could show (or provide a source that indicates) the marker coordinates derived from RepeatMasker is sufficient. In other words, if they could show that limiting the analysis to those regions defined by SRS, as opposed to defining a marker set based on LRS data, will not be missing some repeat regions with a potential to include MSI.

The markers were selected using the human reference genome assembly GRCh38 alone; neither short-read sequencing (SRS) nor long-read sequencing (LRS) data were used during the initial marker selection. We subsequently refined the panel using HPRC LRS data to remove sites with high missingness or evidence of excessive instability. Our goal was to retain an inclusive marker set while remaining within practical limits imposed by alignment accuracy and runtime. Owl is also flexible with respect to marker choice: users can provide alternative marker files, and we include documentation describing how to generate custom marker sets:

https://github.com/PacificBiosciences/owl/blob/main/analysis/MARKERS.md

The following text (149-155) has been revised for clarity:

Owl requires a catalog of repeats in the human reference genome GRCh38 for instability analysis. To generate this reference-based catalog, we curated microsatellite annotations from RepeatMasker tracks available through the UCSC Genome Browser (Kent et al. 2002), we selected simple 1-6 bp repeats spanning 20-100 bp with sequence identity greater than 97%. A stringent motif-to-reference alignment identity cutoff was required because Owl's alignment algorithm was not optimized for divergent or interrupted repeat copies.

Fig 2B vs 2C: In Fig 2B, it appears that SNU-1 and TC32 have the highest MSI in terms of the number of % of instable motifs. However, in Fig 2C and as the authors claim in the text, CHLA10 (not SNU-1) and TC32 have the highest number of instable motifs. Is this a mistake or else, how do the authors justify this seemingly inconsistent result?

Figures 2B and 2C reflect distinct, independent analyses, as they did in the original submission. Figure 2B summarizes the overall MSI percentage, whereas Figure 2C uses a binomial model applied at the motif level to identify motifs that significantly exceed their expected background distribution. Thus, a sample may contain more

categories of significantly unstable motifs, even if those motifs are relatively rare and contribute less to the aggregate MSI percentage.

However, the underlying dataset has changed in the revised manuscript. As noted above, two samples, CHLA10 and TC32, were identified as cross-contaminated and were removed from the analyses, and six additional cell lines were added. These changes affect the composition of the analyzed cohort and therefore the quantitative results shown in the revised figures.

The following text (243-246) has been edited to emphasize the MSI scores are disjoint from the motif analysis:

*Next, we analyzed baseline motif-specific instability across the 131 HPRC controls (Figure 2B). This analysis was independent of the MSI score and modeled which motifs in the cancer genome were instability outliers. We modeled the controls using a hierarchical beta-binomial model that pools counts across samples for each motif and generates posterior-predictive p-values to flag motifs whose observed instability exceeds expectation (**see Methods**).*

Fig 2B vs 2C: Please use consistent identifier for SNU-1 and NCC59 in both panels

(avoid the NPM identifiers).

Thank you for catching this oversight, we've updated the sample names in the Figure 2B/C.

The authors devoted a significant part of the analysis to GGAA motif, therefore, they should highlight GGAA (or its representative) on Fig 2B and 2C.

We have removed discussion of the GGAA finding because these samples were labeled MSI because of cross-contamination. The GGAA motif may have appeared as an outlier due to inter-individual variation at GGAA repeat loci rather than tumor-associated instability.

What was Owl's predictive power (AUROC and/or precision/recall) for detecting MSI based on the literature? While Owl detected five MSI-high samples, it is unclear whether those were the only MSI-high samples or Owl missed some.

We have added an accuracy statement based on a meta-analysis of the available short-read and long-read datasets analyzed with Owl and MSIsensor-pro. We also highlight the single sample that we believe represents an Owl false negative.

Added text:

> Given one false negative in this small sample size we calculate and accuracy for Owl of 90.9%.

The authors compared their LRS MSI calling method to SRS MSI caller (DRAGEN) on only one astrocytoma sample and on only exonic regions. If the authors claim a similar or superior performance of Owl using LRS compared to a SRS MSI caller, they should evaluate more than one sample for which both LRS and SRS are available.

We have extended our comparisons to 11 samples with matched WGS HiFi and Illumina data.

Please reference the new results paragraph comparing Owl to MSIsensor-pro.

Equally importantly, they could examine WGS regions. This is important as they claimed in the Ewing sarcoma example (and possibly other diseases), MSI can occur in non-coding regulatory regions and therefore, limiting the analysis to exonic SRS data may not produce the full picture (whole-genome and whole-exome results may be consistent, but it should be examined).

We have extended our comparisons to 11 samples with matched WGS HiFi and Illumina data.

Please reference the new results paragraph comparing Owl to MSIsensor-pro.

Perhaps, a major limitation of this study is the lack of patient data and solely using LRS on cell lines. Are the MSI rates (%) and thresholds applicable in the context of human primary tumor data? Could the authors perform a similar analysis on a limited panel of patient data, or if not, could they provide information as to how this could be addressed when analyzing patient LRS data? It would significantly strengthen the study if Owl is fine-tuned with or run on patient data.

The reviewer makes a fair point; we have limited patient data. We did repeat the CV parametric model fitting analysis on the matched CaVal13 control, and updated table S3 (measures of parametric model fit). Generally, across hundreds of cell lines, and across two healthy blood samples (NA12878 and CaVal13) we find that the CV distribution is

relatively stable. The global MSI thresholds (10%) and the CV score threshold per marker are all adjustable, and easily analyzed as they are in TSV files.

Added text to the methods section:

Additionally, we fit the same distributions to the CaVal-13 (normal/blood) control dataset and observed similar results. Under the gamma model, the 95th percentile (p = 0.95) corresponds to a CV score of 4.34. Taken together with the cell line analysis, this indicates consistent behavior across both a cell line and a blood sample.

Heavily revised discussion section:

The present Owl MSI analyses are limited by the number of long-read cancer genomes currently available, and the comparisons presented here are restricted to a single primary tumor sample. The Owl global threshold of 10% was empirically defined using cell line data and may require adjustment in larger cancer cohorts. The per-locus CV score threshold, global MSI threshold, and marker set are all adjustable. With a cohort of primary tumors and orthogonal MSI assay results, a logistic classifier could be trained to provide more refined MSS/MSI classification for HiFi data.

The authors should provide the runtime and system (HPC) configurations used to achieve that runtime and performance.

We have added that information at the end of the results section and Table S7. No special hardware was used during these computes, and similar runtimes were found of the authors mac book.

Added text to the results section:

We additionally evaluated runtime using this titration series (Table S7). Runtime increased approximately linearly with sequencing depth, ranging from ~5-9 minutes at 7.5x coverage to ~13-21 minutes at 20x, with low variability across replicates (3-30 seconds). As Owl is not multithreaded, runtimes reflect single-core performance and scale predictably with input size.

Did the authors use only one cell line (NA12878) to define the CV threshold of 5%? Why didn't they use other "normal" cell lines to ensure a consistent CV threshold? This point could be a major limitation and source of variation for real-world LRS clinical data as well as potentially for cell lines. The authors could have a more robust approach of choosing a threshold that does not solely rely on one cell line.

We extended this parametric analysis (**Table S3**) to include the patient control sample (CaVal13-N). We find a similar cutoff compared to NA12878 and think the CV threshold is stable across MSS samples.

Minor points:
L249: "microstalite" typo

Fixed the typo on 249 and 253, thank you for catching it.

L249: "a intronic" to an intronic

Fixed

L480: "Singapore: Waiting for Kie-Kyon to reply" to be removed

The following text has been added:

> **The two gastric cancer cell line samples:**
>
> https://ega-archive.org/datasets/EGAD50000002300

All figures and specifically Fig 2B: font sizes can be bigger for better readability, especially for Fig 2B x-axis and the legend "%high"

We have increased the font size in the main figures (1 and 2) which are now more readable (I too have old eyes). We reviewed the size of the text in the supplemental figures and found them to be sufficiently large.

Supp Fig 5: For a clearer visualization, the authors should show "EZH2" gene name on the x-axis (along with a horizontal line showing this is in an intron) similar to what would have shown if we were looking at this in a genome browser such as IGV.

This figure has been removed because CHLA10 and TC32 were cross contaminated.

A newer revision of ENCODE cCREs have been published in 2026 and the authors could reference the more recent version, should they decide to do so: https://www.nature.com/articles/s41586-025-09909-9

This analysis has been removed because CHLA10 and TC32 were cross contaminated.
