# Supplementary material for: Hunting for microsatellite instability in long-read data with Owl
Source: PLoS Comput Biol. 2026 Sep 2;22(9):e1014423. doi: 10.1371/journal.pcbi.1014423 (PMC13585337; doi:10.1371/journal.pcbi.1014423)
Supplement: Supplementary file 3 [file pcbi.1014423.s012.docx]

# Supplemental Methods

The *Owl* microsatellite instability detection tool was implemented in Rust and provides three subtools: profile, merge, and score, which are described below. The software is public and distributed under [PacBio’s license](https://github.com/PacificBiosciences/owl/blob/main/LICENSE.md) on GitHub: [https://github.com/PacificBiosciences/owl](https://github.com/PacificBiosciences/owl/tree/main/data). Both the source code and pre-compiled static binaries are available. *Owl* has two inputs, an aligned and haplotagged bam file, and a motif BED file containing regions and their motifs, see provided example in the repository (<https://github.com/PacificBiosciences/owl/tree/main/data>). Users may generate their own marker files or reproduce the provided file ([https://github.com/PacificBiosciences/owl/analysis/MARKERS.md](https://github.com/PacificBiosciences/owl/blob/rep_anno/analysis/MARKERS.md)).

During the profiling phase, *Owl* queries the BAM files for genomic regions corresponding to each repeat marker. Alignments fully spanning these regions are filtered and processed. *Owl* should be run on reads that have already been haplotagged by tools like Hiphase [(Holt et al. 2024)](https://paperpile.com/c/K6maFu/i3uN) or WhatsHap [(Patterson et al. 2015)](https://paperpile.com/c/K6maFu/pmfC). For each read, the portion of the read overlapping a tandem repeat is determined by the base-level alignment, and is extracted along with a flanking sequence (10 bp, total) for context.

Local wrap-around dynamic programming (WDP) [(Landau et al. 2001; Myers and Miller 1989)](https://paperpile.com/c/K6maFu/Ghv4+Fv0p) is used to score an alignment of an optimal number of copies of a motif. Briefly, WDP finds the optimal local alignment of a sequence S to T*, the Kleene closure of the motif sequence T (e.g. {NULL, AT, ATAT, ATATAT...} for motif AT). The dynamic programming recursions are defined for regular expression matching [(Myers and Miller 1989; Gusfield 2010)](https://paperpile.com/c/K6maFu/Fv0p+7J6o). The scoring matrices are defined to exclude alignments with fewer than 50% matches. Only the most frequently occurring motif is considered, in contrast to the string decomposition approach used for annotating motif diversity of centromere [(Dvorkina et al. 2020)](https://paperpile.com/c/K6maFu/OMIK) and VNTR sequences [(Ren et al. 2023)](https://paperpile.com/c/K6maFu/jnJV) where motif libraries are considered. The function returns an alignment score and the coordinates of the repeat span within the read, allowing each read to be assigned a single repeat length for that marker. Reads with low alignment scores (<90% identity) are excluded from further analysis.

After annotation, repeat lengths are grouped by their haplotype tag (HP). The mean and standard deviation of repeat lengths are then calculated for each haplotype to summarize copy variation within the sample. While the standard deviation should be sufficient to describe repeat variability, there is a positive relationship with the length of the repeat and the standard deviation. In other words, large repeats tend to have larger outliers (with respect to length), and increased standard deviation. This often happens when a few read alignments place the repeat insertion outside the target interval. To account for this issue, rather than filtering outliers, the standard deviation of repeat length is normalized by the mean repeat length. The coefficient of variation (CV), accounts for larger means having higher variability and allows better comparison across datasets. At the end of the *Owl* profile stage, the program reports the site, motif, and, for each haplotype, the coverage, mean repeat length, coefficient of variation expressed as a percentage (CV), and repeat lengths (when using the --keep-lengths option). The output is a text file modeled after the Variant Call Format (VCF), with a header that defines each field and a structure that supports merging multiple samples into a single file.

To determine an empirical cutoff for the coefficient of variation (CV), the metric used to quantify marker instability, we fitted parametric distributions to a control cell line (NA12878)[(Kronenberg et al. 2025)](https://paperpile.com/c/K6maFu/P4Sh). Using the *fitdistrplus* [R] package [(Delignette-Muller and Dutang 2015)](https://paperpile.com/c/K6maFu/VmXW), normal, gamma, and Weibull distributions were evaluated. Sites with a CV of zero (14%) were excluded, as invariant loci do not capture sequencing error and would require a mixture of models to represent accurately. The upper tail of the distribution was also trimmed at the 99.9th percentile (CV = 20), as extreme values likely reflect alignment artifacts, phasing errors, or incorrect repeat unit identification. Among the models tested, the gamma distribution provided the best fit (shape = 3.4, rate = 1.41) (**S3 Table**), summarized in **S2 Fig**. Quantile-quantile plots showed divergence above a CV of approximately 6, although the empirical and theoretical cumulative distribution functions were stable across the range (**S2 Fig**). Based on this model, a probability threshold of 0.05 corresponds to a CV value of 4.8, rounded up to 5.0 (p = 0.0446). This CV threshold was applied in subsequent analyses and produced consistent, biologically reasonable results across multiple samples. Additionally, we fit the same distributions to the CaVal-13 (normal/blood) control dataset and observed similar results. Under the gamma model, the 95th percentile (p = 0.95) corresponds to a CV score of 4.34. Taken together with the cell line analysis, this indicates consistent behavior across both a cell line and a blood sample.

In the final stage of the process, *Owl* score compares the profiled markers against the defined CV threshold. Each haplotype at a marker site is evaluated, while no-called alleles are excluded from the tally. Every called haplotype contributes to the total number of alleles scored. The Score module reports the number of high- and low-CV alleles and summarizes the overall percentage of high-CV alleles as the genome-wide MSI score. In addition to the MSI score, the output includes the percentage of phased sites and the fraction of markers passing quality thresholds.

### Marker population-level filtering

We downloaded genomic DNA sequencing data for 131 samples from HPRC Data Releases 1 and 2 and downsampled to approximately 30-40x depth. Reads were processed with PacBio WGS Variant Pipeline v3.0.2, including alignments to GRCh38_no_alt_analysis_set, variant calls with DeepVariant v1.9.0 and variant phasing and alignment haplotagging with HiPhase v1.5.0. All HPRC sample names used in this study are listed in **S2** **Table**.

To improve marker reliability, we applied population-level analyses across the HPRC genomes to identify sites that were frequently no-called or consistently scored as MSI in our control samples. Among the 164,374 candidate markers, 32,724 (20%) showed at least one no-call, with a median no-call rate of 13%, and 5,872 sites (3.5%) failed in all samples, typically due to insufficient read support. High-CV events were even more common: 122,453 markers (74%) had at least one sample with CV>5, although these events were generally rare at the population scale, with a median of only 2.29% of samples affected per site and just four markers with high CV in all individuals. Using these population-derived metrics, we removed sites where more than 25% (>32) of samples were either no-called or had high CV, yielding 17,790 unreliable markers that were excluded from the final marker set (146,562) distributed with *Owl*.

### Motif specific instability

We quantified motif-level instability by modeling the control cohort using a hierarchical Beta-Binomial. We’ve provided the code and files (**10.5281/zenodo.18437022**). For each sample and motif, we counted the number of unstable events (high) and the total number of evaluated sites (n = high + low). For each motif m, we pooled counts across all 131 HPRC control samples to obtain:

k_m_ = sum of high counts for motif m
n_m_ = sum of total counts for motif m

To estimate the underlying instability rate for each motif, we assumed:

k_m_ | p_m_ ~ Binomial(n_m_, p_m_)

and placed a Beta prior on p_m_ defined by hyperparameters α and β:

p_m_ ~ Beta(α, β)

The prior mean was set to the global average instability rate across all motifs in the control cohort, and the prior strength controlled the degree of pooling across motifs. The average instability rate was estimated as μ = 0.0297. The prior strength was set as the median number of evaluated sites across samples, which is given by s = median_m_ (n_m_) = 8. The Beta prior parameters are then set as

α = s*μ = 0.2381

β = s*(1 - μ) = 7.761.

Posterior parameters for each motif were computed using the conjugate update:

α_m post_ = α + k_m_
β_m post_ = β + (n_m_ − k_m_).

The posterior mean instability rate for motif m was:

p_m post_ = α_m post_ / (α_m post_ + β_m post_)

To assess whether a cancer sample showed more instability than expected for a given motif, we used the posterior predictive distribution under the control model. For a new sample with observed high count k_new_ and total n_new_ at motif m, the predictive probability of observing k_new_ or more unstable sites was computed from the Beta-Binomial distribution:

P(K ≥ k_new_ | n_new_, α_m post_, β_m post_)

This yields a one-sided posterior-predictive p-value. Motifs whose p-values were small indicate instability greater than expected based on the controls. We applied Bonferroni correction across motifs to control for multiple testing. Under the Bonferroni method, an individual p-value is considered significant if:

p_corrected = p_raw × number_of_tests
and p_corrected < 0.05

We computed these p-values separately for each cancer sample and retained all motifs with statistically significant excess instability.

### DRAGEN MSI analysis

Illumina exome sequencing was performed on an anaplastic astrocytoma tumor (caVal-13) and its matched normal using the IDT xGen Exome Research Panel v2.0. Sequencing was carried out to achieve an average coverage of 300x across targeted regions. Sequencing data were processed with the Illumina DRAGEN v4.0.3 to assess MSI in both tumor-normal and the tumor-only mode. For the tumor-only mode, a panel of normals with 90 samples was used.

### MSIsensor‑pro analysis

Whole genome sequencing reads obtained from the SRA were aligned to the GRCh38 reference genome using bwa‑mem2 v2.3, and the resulting BAM files were analyzed for microsatellite instability (MSI) using MSIsensor‑pro v1.3.0. MSI for these samples was assessed in tumor‑only mode, comparing each tumor BAM to the GRCh38 TCGA baseline (https://github.com/xjtu-omics/msisensor-pro/blob/master/data/GRCh38.baseline_TCGA-v1.3.tsv), with MSI scores defined as the proportion of unstable microsatellite loci among all evaluable sites.

### Titrations and Benchmarking

HiFi sequencing data from two genomes, NA12878 (microsatellite stable, MSS) and NCC-59 (microsatellite instability, MSI), were downsampled to coverage levels ranging from 7.5x to 20x. The datasets were processed using the Human WDL pipeline (<https://github.com/PacificBiosciences/HiFi-human-WGS-WDL>), producing haplotagged BAM files. *Owl* was then applied to quantify global MSI rates (**S6 Table**). Runtime performance of *Owl* was benchmarked using Snakemake v9.12.0 (**S7 Table**) (<https://snakemake.readthedocs.io/en/v9.12.0/tutorial/additional_features.html#benchmarking>).

### HiFi sequencing of six cancer cell lines

SMRTbell libraries were prepared with 5 ug input on a Hamilton NGS STAR MOA system following protocol, “Preparing whole genome libraries using the HiFi prep kit 96” (PN 103-420-700). Polymerase bound SPRQ libraries were sequenced on a Revio system running instrument software v13.3 at a loading concentration of 300 pM with adaptive loading for 24 hours.

### Supplemental References

[Delignette-Muller, Marie Laure, and Christophe Dutang. 2015. “Fitdistrplus: AnRPackage for Fitting Distributions.”](http://paperpile.com/b/K6maFu/VmXW) *[Journal of Statistical Software](http://paperpile.com/b/K6maFu/VmXW)* [64 (4). https://doi.org/](http://paperpile.com/b/K6maFu/VmXW)[10.18637/jss.v064.i04](http://dx.doi.org/10.18637/jss.v064.i04)[.](http://paperpile.com/b/K6maFu/VmXW)

[Dvorkina, Tatiana, Andrey V. Bzikadze, and Pavel A. Pevzner. 2020. “The String Decomposition Problem and Its Applications to Centromere Analysis and Assembly.” *Bioinformatics (Oxford, England)* 36 (Suppl_1): i93–i101.](http://paperpile.com/b/K6maFu/OMIK)

[Gusfield, Dan. 2010. *Algorithms on Strings, Trees, and Sequences*. Cambridge University Press. https://doi.org/](http://paperpile.com/b/K6maFu/7J6o)[10.1017/cbo9780511574931](http://dx.doi.org/10.1017/cbo9780511574931)[.](http://paperpile.com/b/K6maFu/7J6o)

[Holt, James M., Christopher T. Saunders, William J. Rowell, Zev Kronenberg, Aaron M. Wenger, and Michael Eberle. 2024. “HiPhase: Jointly Phasing Small, Structural, and Tandem Repeat Variants from HiFi Sequencing.” *Bioinformatics (Oxford, England)* 40 (2). https://doi.org/](http://paperpile.com/b/K6maFu/i3uN)[10.1093/bioinformatics/btae042](http://dx.doi.org/10.1093/bioinformatics/btae042)[.](http://paperpile.com/b/K6maFu/i3uN)

[Kronenberg, Zev, Cillian Nolan, David Porubsky, et al. 2025. “The Platinum Pedigree: A Long-Read Benchmark for Genetic Variants.” *Nature Methods* 22 (8): 1669–1676.](http://paperpile.com/b/K6maFu/P4Sh)

[Landau, G. M., J. P. Schmidt, and D. Sokol. 2001. “An Algorithm for Approximate Tandem Repeats.” *Journal of Computational Biology: A Journal of Computational Molecular Cell Biology* 8 (1): 1–18.](http://paperpile.com/b/K6maFu/Ghv4)

[Myers, E. W., and W. Miller. 1989. “Approximate Matching of Regular Expressions.” *Bulletin of Mathematical Biology* 51 (1): 5–37.](http://paperpile.com/b/K6maFu/Fv0p)

[Patterson, Murray, Tobias Marschall, Nadia Pisanti, et al. 2015. “WhatsHap: Weighted Haplotype Assembly for Future-Generation Sequencing Reads.” *Journal of Computational Biology: A Journal of Computational Molecular Cell Biology* 22 (6): 498–509.](http://paperpile.com/b/K6maFu/pmfC)

[Ren, Jingwen, Bida Gu, and Mark J. P. Chaisson. 2023. “Vamos: Variable-Number Tandem Repeats Annotation Using Efficient Motif Sets.” *Genome Biology* 24 (1): 175.](http://paperpile.com/b/K6maFu/jnJV)
