# Supplementary material for: Hunting for microsatellite instability in long-read data with Owl
Source: PLoS Comput Biol. 2026 Sep 2;22(9):e1014423. doi: 10.1371/journal.pcbi.1014423 (PMC13585337; doi:10.1371/journal.pcbi.1014423)
Supplement: Peer review history

# Peer review: Decision Letter 0

**DOI:** 10.1371/journal.pcbi.1014423.r001

**Authors:** Can Yang, Ruslan Kalendar

1 Apr 2026

PCOMPBIOL-D-26-00340

Hunting for microsatellite instability in long-read data with Owl

PLOS Computational Biology

Dear Dr. Kronenberg,

Thank you for submitting your manuscript to PLOS Computational Biology. After careful consideration, we feel that it has merit but does not fully meet PLOS Computational Biology's publication criteria as it currently stands. Therefore, we invite you to submit a revised version of the manuscript that addresses the points raised during the review process.

Please submit your revised manuscript by Jun 01 2026 11:59PM. If you will need more time than this to complete your revisions, please reply to this message or contact the journal office at ploscompbiol@plos.org. When you're ready to submit your revision, log on to https://www.editorialmanager.com/pcompbiol/ and select the 'Submissions Needing Revision' folder to locate your manuscript file.

Please include the following items when submitting your revised manuscript:

* A letter that responds to each point raised by the editor and reviewer(s). You should upload this letter as a separate file labeled 'Response to Reviewers'. This file does not need to include responses to formatting updates and technical items listed in the 'Journal Requirements' section below.

* A marked-up copy of your manuscript that highlights changes made to the original version. You should upload this as a separate file labeled 'Revised Manuscript with Track Changes'.

* An unmarked version of your revised paper without tracked changes. You should upload this as a separate file labeled 'Manuscript'.

If you would like to make changes to your financial disclosure, competing interests statement, or data availability statement, please make these updates within the submission form at the time of resubmission. Guidelines for resubmitting your figure files are available below the reviewer comments at the end of this letter.

We look forward to receiving your revised manuscript.

Kind regards,

Ruslan Kalendar, PhD

Academic Editor

PLOS Computational Biology

Can Yang

Section Editor

PLOS Computational Biology

**Additional Editor Comments:**

Authors are required to take the reviewers’ comments into account and revise their articles accordingly.

**Journal Requirements:**

If the reviewer comments include a recommendation to cite specific previously published works, please review and evaluate these publications to determine whether they are relevant and should be cited. There is no requirement to cite these works unless the editor has indicated otherwise.

1) We ask that a manuscript source file is provided at Revision. Please upload your manuscript file as a .doc, .docx, .rtf or .tex. If you are providing a .tex file, please upload it under the item type u2018LaTeX Source Fileu2019 and leave your .pdf version as the item type u2018Manuscriptu2019.

2) Please provide an Author Summary. This should appear in your manuscript between the Abstract (if applicable) and the Introduction, and should be 150-200 words long. The aim should be to make your findings accessible to a wide audience that includes both scientists and non-scientists. Sample summaries can be found on our website under Submission Guidelines:

https://journals.plos.org/ploscompbiol/s/submission-guidelines#loc-parts-of-a-submission

3) Your manuscript is missing the following sections: Design and Implementation, Results, and Availability and Future Directions. Please ensure that your article adheres to the standard Software article layout and order of Abstract, Introduction, Design and Implementation, Results, and Availability and Future Directions. For details on what each section should contain, see our Software article guidelines:

https://journals.plos.org/ploscompbiol/s/submission-guidelines#loc-software-submissions

4) Please upload all main figures as separate Figure files in .tif or .eps format. For more information about how to convert and format your figure files please see our guidelines:

https://journals.plos.org/ploscompbiol/s/figures

5) We have noticed that you have uploaded Supporting Information files, but you have not included a list of legends. Please add a full list of legends for your Supporting Information files after the references list.

6) Please include the Data and Code Availability statement and the Competing Interests statement in the online form.

7) Some material included in your submission may be copyrighted. According to PLOS's copyright policy, authors who use figures or other material (e.g., graphics, clipart, maps) from another author or copyright holder must demonstrate or obtain permission to publish this material under the Creative Commons Attribution 4.0 International (CC BY 4.0) License used by PLOS journals. Please closely review the details of PLOS's copyright requirements here: PLOS Licenses and Copyright. If you need to request permissions from a copyright holder, you may use the PLOS Content Copyright Permission Form Please respond directly to this email and provide any known details concerning your material's license terms and permissions required for reuse, even if you have not yet obtained copyright permissions or are unsure of your material's copyright compatibility. Once you have responded and addressed all other outstanding technical requirements, you may resubmit your manuscript within Editorial Manager. Potential Copyright Issues:

i) Figure 1A. Please confirm whether you drew the images / clip-art within the figure panels by hand. If you did not draw the images, please provide (a) a link to the source of the images or icons and their license / terms of use; or (b) written permission from the copyright holder to publish the images or icons under our CC BY 4.0 license. Alternatively, you may replace the images with open source alternatives. See these open source resources you may use to replace images / clip-art: - https://commons.wikimedia.org - https://openclipart.org/.

**Reviewers' comments:**

Reviewer's Responses to Questions

**Comments to the Authors:**

**Please note here if the review is uploaded as an attachment.**

Reviewer #1:

please refer Question to author section in attachment.

Reviewer #2:

The manuscript introduces Owl, a computational method for identifying microsatellite instability (MSI) with long read sequencing (PacBio) data. The proposed method is well motivated, and the use of haplotype resolved reads provides advantage to traditional short read methods. In addition, the motif-level and regulatory insights enabled by this framework highlight the added value of long-read sequencing beyond conventional MSI classification. Overall, this method is a valuable contribution to current bioinformatics tools for long-read data.

A few minor revisions would strengthen the validation of the tool:

1. The benchmarking is limited to small set of samples against few tools. Although it is discussed that conventional MSI assays on homopolymers might misclassify sarcoma samples with elevated GGAA repeat instability, it would be great to include a figure panel to illustrate cases where Owl outperforms established tools (if short read sequencing data is available on the same samples) and methods (for example, using canonical marker panels only).

2. Since the method relies on phasing, a breif discussion of how lower sequencing depth and reduced phasing quality affect results would increase the clarity. A comment on applicability to other long-read platforms would be helpful.

3. In line 405-413, parameters (alpha_m, beta_m, k_m and n_m) should be reformatted as subscripts for consistency.

Reviewer #3:

Kronenberg et al created a tool called Owl for detecting microsatellite instabilities using long read sequencing data. While Owl was used over a diverse panel of cell lines and reported some useful results, there are several limitations and unclear design choices. There also appear to be some inconsistencies between Fig 2B and 2C.

Major points:

The set of marker coordinates was derived from RepeatMasker which I think must have been obtained from short read sequencing (SRS). It appears that Owl limits its analysis to those regions obtained by SRS, while in theory it might use the full power of long read sequencing (LRS). It would be interesting if the authors could show (or provide a source that indicates) the marker coordinates derived from RepeatMasker is sufficient. In other words, if they could show that limiting the analysis to those regions defined by SRS, as opposed to defining a marker set based on LRS data, will not be missing some repeat regions with a potential to include MSI.

Fig 2B vs 2C: In Fig 2B, it appears that SNU-1 and TC32 have the highest MSI in terms of the number of % of instable motifs. However, in Fig 2C and as the authors claim in the text, CHLA10 (not SNU-1) and TC32 have the highest number of instable motifs. Is this a mistake or else, how do the authors justify this seemingly inconsistent result?

Fig 2B vs 2C: Please use consistent identifier for SNU-1 and NCC59 in both panels (avoid the NPM identifiers).

The authors devoted a significant part of the analysis to GGAA motif, therefore, they should highlight GGAA (or its representative) on Fig 2B and 2C.

What was Owl’s predictive power (AUROC and/or precision/recall) for detecting MSI based on the literature? While Owl detected five MSI-high samples, it is unclear whether those were the only MSI-high samples or Owl missed some.

The authors compared their LRS MSI calling method to SRS MSI caller (DRAGEN) on only one astrocytoma sample and on only exonic regions. If the authors claim a similar or superior performance of Owl using LRS compared to a SRS MSI caller, they should evaluate more than one sample for which both LRS and SRS are available.

Equally importantly, they could examine WGS regions. This is important as they claimed in the Ewing sarcoma example (and possibly other diseases), MSI can occur in non-coding regulatory regions and therefore, limiting the analysis to exonic SRS data may not produce the full picture (whole-genome and whole-exome results may be consistent, but it should be examined).

Perhaps, a major limitation of this study is the lack of patient data and solely using LRS on cell lines. Are the MSI rates (%) and thresholds applicable in the context of human primary tumor data? Could the authors perform a similar analysis on a limited panel of patient data, or if not, could they provide information as to how this could be addressed when analyzing patient LRS data? It would significantly strengthen the study if Owl is fine-tuned with or run on patient data.

The authors should provide the runtime and system (HPC) configurations used to achieve that runtime and performance.

Did the authors use only one cell line (NA12878) to define the CV threshold of 5%? Why didn’t they use other “normal” cell lines to ensure a consistent CV threshold? This point could be a major limitation and source of variation for real-world LRS clinical data as well as potentially for cell lines. The authors could have a more robust approach of choosing a threshold that does not solely rely on one cell line.

Minor points:

L249: “microstalite” typo

L249: “a intronic” to an intronic

L480: “Singapore: Waiting for Kie-Kyon to reply” to be removed

All figures and specifically Fig 2B: font sizes can be bigger for better readability, especially for Fig 2B x-axis and the legend “%high”

Supp Fig 5: For a clearer visualization, the authors should show “EZH2” gene name on the x-axis (along with a horizontal line showing this is in an intron) similar to what would have shown if we were looking at this in a genome browser such as IGV.

A newer revision of ENCODE cCREs have been published in 2026 and the authors could reference the more recent version, should they decide to do so: https://www.nature.com/articles/s41586-025-09909-9

**********

**Have the authors made all data and (if applicable) computational code underlying the findings in their manuscript fully available?**

The PLOS Data policy requires authors to make all data and code underlying the findings described in their manuscript fully available without restriction, with rare exception (please refer to the Data Availability Statement in the manuscript PDF file). The data and code should be provided as part of the manuscript or its supporting information, or deposited to a public repository. For example, in addition to summary statistics, the data points behind means, medians and variance measures should be available. If there are restrictions on publicly sharing data or code —e.g. participant privacy or use of data from a third party—those must be specified.

Reviewer #1: **No:** Data availability from Singapore gastric cancer samples is incomplete.

Reviewer #2: Yes

Reviewer #3: Yes

**********

PLOS authors have the option to publish the peer review history of their article (what does this mean?). If published, this will include your full peer review and any attached files.

If you choose “no”, your identity will remain anonymous but your review may still be made public.

**Do you want your identity to be public for this peer review?**  For information about this choice, including consent withdrawal, please see our Privacy Policy.

Reviewer #1: No

Reviewer #2: No

Reviewer #3: No

[NOTE: If reviewer comments were submitted as an attachment file, they will be attached to this email and accessible via the submission site. Please log into your account, locate the manuscript record, and check for the action link "View Attachments". If this link does not appear, there are no attachment files.]

**Figure resubmission:**

While revising your submission, we strongly recommend that you use PLOS’s NAAS tool (https://ngplosjournals.pagemajik.ai/artanalysis) to test your figure files. NAAS can convert your figure files to the TIFF file type and meet basic requirements (such as print size, resolution), or provide you with a report on issues that do not meet our requirements and that NAAS cannot fix.

After uploading your figures to PLOS’s NAAS tool - https://ngplosjournals.pagemajik.ai/artanalysis, NAAS will process the files provided and display the results in the "Uploaded Files" section of the page as the processing is complete. If the uploaded figures meet our requirements (or NAAS is able to fix the files to meet our requirements), the figure will be marked as "fixed" above. If NAAS is unable to fix the files, a red "failed" label will appear above. When NAAS has confirmed that the figure files meet our requirements, please download the file via the download option, and include these NAAS processed figure files when submitting your revised manuscript.

**Reproducibility:**

To enhance the reproducibility of your results, we recommend that authors of applicable studies deposit laboratory protocols in protocols.io, where a protocol can be assigned its own identifier (DOI) such that it can be cited independently in the future. Additionally, PLOS ONE offers an option to publish peer-reviewed clinical study protocols. Read more information on sharing protocols at https://plos.org/protocols?utm_medium=editorial-email&utm_source=authorletters&utm_campaign=protocols

Attachment Submitted filename: Myreview for owl.pdf

# Author comment: Author response to Decision Letter 1

**DOI:** 10.1371/journal.pcbi.1014423.r002

1 Jun 2026

Attachment Submitted filename: response-to-reviewers.pdf

# Editor report: Decision Letter 1

**DOI:** 10.1371/journal.pcbi.1014423.r003

**Authors:** Can Yang, Ruslan Kalendar

7 Jun 2026

Dear Mr. Kronenberg,

We are pleased to inform you that your manuscript 'Hunting for microsatellite instability in long-read data with Owl' has been provisionally accepted for publication in PLOS Computational Biology.

Before your manuscript can be formally accepted you will need to complete some formatting changes, which you will receive in a follow up email. A member of our team will be in touch with a set of requests.

Please note that your manuscript will not be scheduled for publication until you have made the required changes, so a swift response is appreciated.

IMPORTANT: The editorial review process is now complete. PLOS will only permit corrections to spelling, formatting or significant scientific errors from this point onwards. Requests for major changes, or any which affect the scientific understanding of your work, will cause delays to the publication date of your manuscript.

Should you, your institution's press office or the journal office choose to press release your paper, you will automatically be opted out of early publication. We ask that you notify us now if you or your institution is planning to press release the article. All press must be co-ordinated with PLOS.

Thank you again for supporting Open Access publishing; we are looking forward to publishing your work in PLOS Computational Biology.

Best regards,

Ruslan Kalendar, PhD

Academic Editor

PLOS Computational Biology

Can Yang

Section Editor

PLOS Computational Biology

# Editor report: Acceptance letter

**DOI:** 10.1371/journal.pcbi.1014423.r004

**Authors:** Can Yang, Ruslan Kalendar

PCOMPBIOL-D-26-00340R1

Hunting for microsatellite instability in long-read data with Owl

Dear Dr Kronenberg,

I am pleased to inform you that your manuscript has been formally accepted for publication in PLOS Computational Biology. Your manuscript is now with our production department and you will be notified of the publication date in due course.

The corresponding author will soon be receiving a typeset proof for review, to ensure errors have not been introduced during production. Please review the PDF proof of your manuscript carefully, as this is the last chance to correct any errors. Please note that major changes, or those which affect the scientific understanding of the work, will likely cause delays to the publication date of your manuscript.

Soon after your final files are uploaded, unless you have opted out, the early version of your manuscript will be published online. The date of the early version will be your article's publication date. The final article will be published to the same URL, and all versions of the paper will be accessible to readers.

For Research, Software, and Methods articles, you will receive an invoice from PLOS for your publication fee after your manuscript has reached the completed accept phase. If you receive an email requesting payment before acceptance or for any other service, this may be a phishing scheme. Learn how to identify phishing emails and protect your accounts at https://explore.plos.org/phishing.

Thank you again for supporting PLOS Computational Biology and open-access publishing. We are looking forward to publishing your work!

With kind regards,

Anitha Samidurai

PLOS Computational Biology | Carlyle House, Carlyle Road, Cambridge CB4 3DN | United Kingdom ploscompbiol@plos.org | Phone +44 (0) 1223-442824 | ploscompbiol.org | @PLOSCompBiol
